# Follow-up and risk factors of pregnant women with gestational diabetes in Morocco

**DOI:** 10.4102/jphia.v16i1.1313

**Published:** 2025-08-25

**Authors:** Rachida Boutiti, Hicham Gougueni, Youssef Bouchriti, Abderrahman Arechkik, Safiya Mahlaq, Hayat Iziki, Amina Barkat

**Affiliations:** 1 Health and Nutrition of the Mother and Child Couple Research Team, Faculty of Medicine and Pharmacy, Mohamed V. University, Rabat, Morocco; 2 Department of Midwifery, High Institute of Nursing Professions and Health Techniques, Agadir, Morocco; 3 Department of Geosciences, Environment and Geomatics Laboratory, Faculty of Sciences, University of Ibn Zohr, Agadir, Morocco; 4 Department of Nursing Care, High Institute of Nursing Professions and Health Techniques, Agadir, Morocco; 5 Department of Health Technique, High Institute of Nursing Professions and Health Techniques, Agadir, Morocco; 6 Laboratory of Biostatistics, Clinical Research and Epidemiology (LBRCE), Faculty of Medicine and Pharmacy, Mohamed V. University, Rabat, Morocco

**Keywords:** pregnant women, gestational diabetes mellitus, management, complications, risk factors

## Abstract

**Background:**

Gestational diabetes mellitus (GDM) represents a significant global public health challenge, posing substantial risks to both maternal and foetal health.

**Aim:**

This study analysed risk factors, follow-up, management approaches and obstetric complications in pregnant women with GDM.

**Setting:**

This study was conducted in Southern Morocco.

**Methods:**

This multicentre retrospective cross-sectional study analysed 297 patient files (*n* = 120 with GDM) from four maternity hospitals (2019-2023). Data were collected via a standardised form. Statistical analysis included descriptive summaries, group comparisons (Chi-square, Fisher’s *t*-test, Mann–Whitney *U*) and logistic regression to calculate odds ratios (ORs) using SPSS version 27.

**Results:**

Gestational diabetes mellitus screening was inconsistent: only 17% (95% confidence interval [CI]: 11.5% – 25.6%) of women were screened before 24 weeks of amenorrhea, and 38% (95% CI: 29.8% – 47.5%) between 24 weeks and 28 weeks. The oral glucose tolerance test 75 g was not used. Gestational diabetes mellitus was significantly associated with caesarean delivery (OR = 2.52; 95% CI: 1.29–4.92; *p* = 0.007 and preeclampsia 5.95 (95% CI: 1.21–29.21; *p* = 0.028). Risk factors for GDM included maternal age over 35 years and obesity (body mass index [BMI]: ≥ 30). A history of prematurity showed a significant association with GDM, with adjuster OR (aOR) of 3.47 (95% CI: 1.36–8.79; *p* < 0.011).

**Conclusion:**

Preventing maternal complications from GDM relies on raising women’s awareness about the importance of screening and monitoring during pregnancy in Southern Morocco.

**Contribution:**

This study highlights the necessity of strengthening GDM screening and targeted management strategies for at-risk pregnant women in Morocco, particularly in the southern region.

## Introduction

Gestational diabetes mellitus (GDM) continues to be a global public health issue because of its high prevalence and the impact it has on both mothers and their babies.^1^ It is one of the serious complications during pregnancy. In fact, GDM is becoming an increasing concern worldwide, affecting approximately 7% of all pregnancies.^2^

In Africa, the prevalence is reported to be 13%,^3^ whereas in Morocco, it stands at 8.2%.^4^

Genetic, demographic and sociocultural variables are linked to the development of GDM.^5^ This is further compounded by lifestyle, socioeconomic pressures, decreased physical activity and an overweight body mass index (BMI) of more than 25 kg/m^2^.^6^

From a different perspective, GDM is associated with various maternal complications, including an elevated risk of preeclampsia, difficult labour, the need for instrumental assistance, severe perineal tears and postpartum haemorrhage (PPH).^7^ In addition, there is a higher likelihood of caesarean delivery in cases of GDM, regardless of the type of treatment or the baby’s birth weight.^8^ Effective management of pregnant women with GDM is essential for mitigating the risks associated with this condition for both the mother and her newborn by ensuring that maternal blood glucose levels are normalised.^9^

To address this issue, the American Diabetes Association (ADA), the World Health Organization (WHO), the International Federation of Gynecology and Obstetrics and the Endocrine Society recommend using the criteria established by the International Association of Diabetes and Pregnancy Study Groups (IADPSG) for GDM screening.^10^ The IADPSG guidelines suggest that early screening conducted before 24 weeks of amenorrhea (WA) can identify women who may have type 2 diabetes mellitus prior to becoming pregnant. However, a new screening test between 24 weeks and 28 weeks of WA is indicated for women with normal blood glucose levels (< 0.92 g/L).

Notably, the majority of women (70% – 85%) with GDM can be successfully treated with a healthy lifestyle, proper exercise and dietary modifications.^11^ More than 15% to 30% of these women usually do not need insulin therapy.^12^ Furthermore, a caesarean section is indicated for gestational diabetes because of the increased risk of shoulder dystocia and brachial plexus paralysis.^8,13^

After childbirth, especially during the immediate postpartum period, the management of women involves stopping insulin therapy and recommending fasting blood glucose tests to check for persistent hyperglycaemia. If the fasting plasma glucose level is ≥ 126 mg/dL or the postprandial level is ≥ 200 mg/dL, this indicates confirmed persistent hyperglycaemia and insulin can be prescribed during the postpartum period, even for breastfeeding mothers without concerns about neonatal side effects.^14^ Endocrine follow-up is essential, especially for women who received high doses of insulin during pregnancy.^8^

In Morocco, new guidelines have been introduced recommending a specialised consultation along with a check-up of fasting blood glucose and glycated haemoglobin (or HbA1c) at three months postpartum. Following this, fasting plasma glucose screening should be conducted during the third postpartum consultation, prior to any subsequent pregnancies, and then every 1 year to 3 years based on risk factors, for a minimum of 25 years.^15^

The aim of this study was to investigate the risk factors, therapeutic and obstetrical follow-up of pregnant women with GDM in the Souss-Massa region of southern Morocco.

## Research methods and design

### Study type

Between May 2024 and July 2024, a cross-sectional multicentre study was carried out at four maternity hospitals in the Souss-Massa region of southern Morocco. This study was conducted in accordance with the Strengthening the Reporting of Observational Studies in Epidemiology (STROBE) guidelines relating to the design of cross-sectional studies.^16^

### Study area

Four maternity hospitals in the Souss-Massa region were selected for the study: the regional maternity hospital at Hassan II Hospital in Agadir, the provincial maternity hospital in Inezgane Ait Melloul, the provincial maternity hospital at El Mouhtar Essoussi in Chtouka Ait Baha and the provincial maternity hospital at El Moukhtar Essoussi in Taroudant.

### Target population and sampling

The study examined the delivery records of all pregnant women with GDM who delivered in these four hospital maternity units between January 2019 and December 2023. The delivery records of pregnant women with GDM who delivered at these four maternity hospitals were included in this study. However, delivery records of women with type I and type II diabetes prior to pregnancy, delivery records of women with other pathologies during pregnancy and delivery records with missing or empty data were excluded from this study.

### Sample and sampling technique

The sampling technique used was a non-random sample stratified by quota (by province and by year) at the level of the four maternity units. The sample size was calculated based on a 2.5% margin of error and a 95% confidence interval (CI). This calculation considered the total population of live births in the Souss-Massa region, which amounted to 34 377 births recorded from 2017 to 2021, according to the Regional Health Observatory (2023).

We retained a prevalence of 23.7% based on a Moroccan study carried out in 2018 in the Marrakech Al Haouz region.

The calculation was performed on the sample size calculator website: OpenEpi (version 2013) (https://www.openepi.com/SampleSize/SSPropor.htm).

This number is broken down by province, taking into account the percentage of its population in relation to that of the region.

**FIGURE 1:**
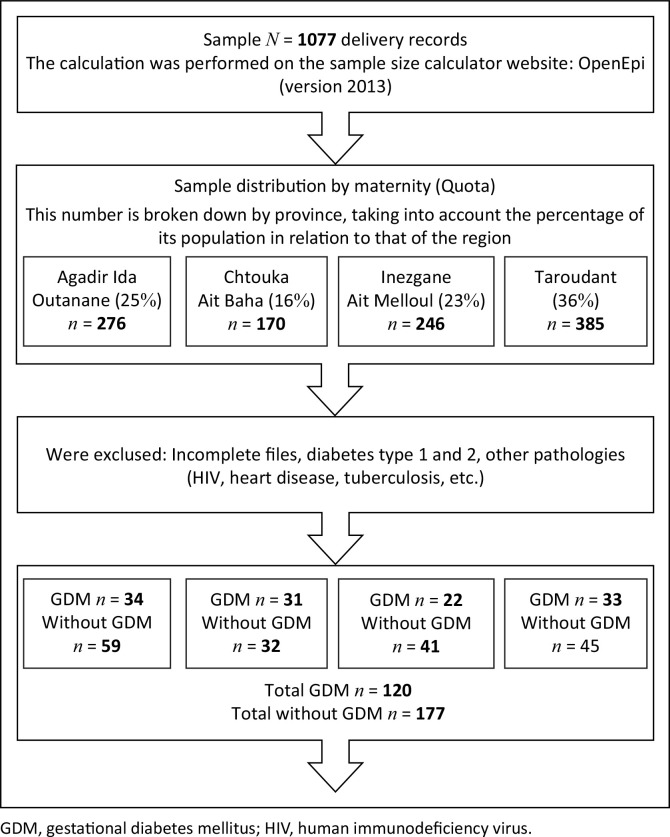
Schematic diagram of study population.

The study ultimately analysed 120 delivery records from women with GDM and 177 records from women without GDM after excluding incomplete or records meeting other exclusion criteria (Figure 1).

### Study variables

#### Sociodemographic characteristics

This includes woman’s age, gravidity (total number of pregnancies), parity (number of live births), marital status and maternal origin.

#### Obstetrical history

This section focused on the history of prematurity, history of macrosomia, history of stillbirths, history of deaths in utero, history of gestational diabetes, history of abortion and history of congenital malformations.

#### History of surgery

Appendectomy, gall bladder, abdominal hernia and cholecystectomy.

#### Medical history

This involves family history of diabetes, chronic maternal morbidity: asthma, anaemia and allergies.

#### Current pregnancy follow-up

This covers aspects such as gestational age, weight (kg), height (cm), uterine height (cm), whether or not the pregnancy is ongoing, whether the pregnancy is classified as normal or at-risk and details of women with GDM who are on insulin treatment or a diet.

#### Specific screening modalities used during pregnancy

The study specifically examined: (1) the practice of early GDM screening (before 24 weeks of WA), (2) the timing of systematic screening (between 24 weeks and 28 weeks of WA) and (3) the gestational age at which pregnancy was terminated because of poor glycaemic control (analysis considered terms such as < 34 weeks, 34 weeks, 36 weeks and 39 weeks of WA).

#### Screening methods

Oral glucose tolerance test (75 g OGTT), fasting blood glucose and glycated haemoglobin (Hb1Ac).

#### Immediate postpartum interventions

This involves aspects such as follow-up by an endocrinologist, decision on insulin therapy (discontinuation or resumption), guidance on capillary blood glucose self-monitoring and dietary advice.

The protocol specified that before hospital discharge, patients should be referred to an endocrinologist. For post-discharge monitoring, a follow-up testing schedule is recommended: a fasting blood glucose test during a late postnatal consultation, a glycated haemoglobin (HbA1c) at three months, and a 75 g oral glucose tolerance test (OGTT) to be performed at four weeks, six weeks and three months postpartum.

#### Obstetric management of the woman

This includes the induction of labour and the reasons for it. During labour, information is gathered on possible complications such as premature rupture of membranes (PROM), placenta previa and placental abruption, as well as other complications such as gestational hypertension, maternal fever, chorioamnionitis, amniotic fluid staining and other problems.

#### Types of delivery

The types of delivery were categorised as normal, premature or scheduled.

#### Mode of delivery

The modes of delivery investigated included vacuum, extraction, episiotomy, scheduled caesarean and emergency caesarean.

#### Caesarean section reasons

The reasons recorded for performing a caesarean section included macrosomia, preeclampsia, poorly controlled GDM, hydramnios, foetal heart rate abnormality, gestational hypertension or other documented reasons.

#### Types of dystocia at the time of delivery

The types of dystocia documented at the time of delivery included tight circular umbilical cord, shoulder dystocia and various forms of perineal trauma, (episiotomy, perineal tear, first-degree tear, second-degree tear or uncomplicated complete perineal tear), as well as cases with an intact perineum.

### Statistical data processing methods

Qualitative variables were summarised using frequencies and percentages. Quantitative variables were described using mean ± standard deviation (s.d.) for normally distributed data or median (interquartile range [IQR]) for non-normally distributed data.

Bivariate analysis was performed using the Chi-square test or Fisher’s exact test for qualitative variables and Student’s *t*-test or Mann–Whitney *U* test for quantitative variables, as appropriate. Adjusted odds ratios (aOR) with 95% CI were calculated to quantify the relative risk of adverse outcomes associated with GDM (compared to women without GDM). In order to analyse the association between GDM and complications while controlling for potential confounders, multivariate analysis was performed using multiple logistic regression models. *P*-values below 0.05 were considered statistically significant. All data were processed using SPSS^®^ version 27.

### Ethical considerations

Anonymity and confidentiality in the processing of data extracted from delivery records were upheld in accordance with the Declaration of Helsinki and its subsequent amendments. Authorisation for data collection was secured from the Direction Régionale de la Santé et de la Protection Sociale (Regional Health and Social Protection Directorate) to access delivery records and conduct this study in hospital maternity units. In addition, ethical approval was granted by the Ethics and Biomedical Research Commission of the Mohammed V University Faculty of Medicine and Pharmacy in Rabat (Reference: 78/24).

## Results

The average age of women with GDM was 33 years, with a s.d. of 6.36 years. The majority of women (60%) came from rural areas. Women with GDM have a higher BMI than women without GDM (the majority are in the ≥ 30 category). The results show that only 11.29% of women with GDM have a normal BMI, while overweight and obesity, corresponding to BMIs > 25 or > 30, are observed in 40% and 54% of participants, respectively.

Women with gestational diabetes showed a significantly higher rate of prenatal monitoring compared to women without gestational diabetes (59.18% vs. 40.82%). Similarly, women with gestational diabetes experienced a higher prevalence of gestational hypertension (66.67%) and preeclampsia (81.25%). Women with GDM have a longer hospital stay than women without GDM.

The picture reveals significant gaps in the management of women with postpartum GDM. Only 10.83% of women are referred to an endocrinologist. Postpartum blood glucose monitoring (fasting and postprandial) is very low. HbA1c testing is carried out in only 5% of women.

Figure 3 reveals significant gaps in the follow-up of women with postpartum GDM. Only 10.83% of patients are followed by an endocrinologist. Similarly, the use of postpartum (FBG_PostPartum) and postprandial (FBG_PostPrandial) monitoring is very low. HbA1c testing is carried out in 5% of women.

Table 2 highlights several statistically significant differences in the course and outcomes of labour and delivery between women with GDM and the control group.

### Increased intrapartum risks

Women with GDM had significantly higher odds of developing pregnancy-induced hypertension during labour (aOR = 3.4; *p* = 0.002).

### Higher caesarean rates and indications

The odds of caesarean delivery were substantially elevated in the GDM group (aOR = 3.4; *p* < 0.001). Consequently, this lowered the odds of spontaneous vaginal birth (aOR = 0.5; *p* = 0.003). This was largely driven by significantly higher odds of caesarean performed for macrosomia (aOR = 6.0; *p* < 0.001) and preeclampsia (aOR = 7.1; *p* = 0.002).

### Increased delivery complications

Gestational diabetes mellitus was associated with a dramatically increased risk of shoulder dystocia (aOR = 12.2; *p* < 0.001) and a higher risk of perineal tears (aOR = 2.6; *p* = 0.04).

### Episiotomy

Conversely women with GDM had lower odds of receiving an episiotomy (aOR = 0.5; *p* = 0.01).

Table 3 presents findings from a multivariate logistic regression analysis evaluating the association between GDM and the occurrence of various obstetric complications. The associations for specific complications were as follows.

### Intrapartum complications

Gestational diabetes mellitus showed a trend towards increased odds (OR = 1.37; 95% CI: 0.70–2.69), but this association did not reach statistical significance (*p* = 0.352).

### Preeclampsia

Gestational diabetes mellitus was strongly and significantly associated with an increased risk of preeclampsia (OR = 5.95; 95% CI: 1.21–29.22; *p* = 0.028).

### Shoulder dystocia

A significant association was observed between GDM and shoulder dystocia (OR = 16.30; 95% CI: 3.32–79.95; *p* = 0.001).

### Perineal tear

Gestational diabetes mellitus was significantly associated with increased odds of perineal tear (OR = 3.037; 95% CI: 1.75–5.28; *p* < 0.001), with odds approximately three times higher for women with GDM.

### Immediate postpartum haemorrhage

A trend towards increased odds of immediate PPH was noted with GDM (OR = 1.82; 95% CI: 0.66–5.01), but this association was not statistically significant (*p* = 0.25).

### Puerperal infection

The association between GDM and puerperal infection was not statistically significant in this analysis (OR = 1.59; 95% CI: 0.68–3.73; *p* = 0.284).

### Medical history

Women with a history of gestational hypertension had a significantly higher risk of being diagnosed with gestational diabetes (aOR = 8.29; *p* < 0.001). Women with a family history of type 2 diabetes also had a significantly higher risk of being diagnosed with GDM (aOR = 21.38; *p* < 0.001).

### History of surgery

Although there was a slight increase in risk for women who had undergone an appendectomy (aOR = 3.52), the *p*-value was not significant (*p* = 0.1069). There was no clear link between appendectomy and diagnosis. Women who underwent gallbladder surgery had a significantly higher risk of being diagnosed (aOR = 2.47; *p* = 0.0627).

### Obstetrical history

Women with a history of abortion had a significantly higher risk of being diagnosed with GDM (aOR = 2.19; *p* = 0.0089). On the other hand, there was no significant association between a history of foetal death in utero and the diagnosis of GDM (aOR = 2.31; *p* = 0.1878). Similarly, there was no significant association between a history of stillbirth and GDM (*p* = 1.0000).

Women with a history of prematurity and macrosomia have a significantly higher risk of being diagnosed with GDM, respectively (aOR = 3.47; *p* = 0.0113), (aOR = 8.92; *p* = 0.0002).

### Nutritional status

Body mass index appears to play an important role in diagnosis. Obese women have a significantly increased risk, while women of normal weight have a reduced risk.

According to the multivariate logistic regression analysis in this table, advancing maternal age was highly significantly associated with increased odds of GDM (*p* < 0.001). Each additional year of age corresponded to a 7.9% increase in odds (OR = 1.08; 95% CI: 1.03–1.13).

Overweight (OR = 0.080) and obesity (OR = 0.34) were associated with marked risk reductions (92% and 66%, respectively). Similarly, family history showed a significant negative association (OR = 0.053; *p* = 0.013), corresponding to a 94.7% risk reduction.

### Obstetric history reveals notable variations

Preterm birth shows β = –1.08 (OR = 0.340; *p* = 0.044), corresponding to a 66% risk reduction. Macrosomia demonstrates β = –1.43 (OR = 0.24; *p* = 0.052), equivalent to a 76% reduction; history of abortion shows a non-significant β (–0.105; *p* = 0.765).

## Discussion

### Managing therapeutic gestational diabetes mellitus during pregnancy, childbirth and the postpartum period

This study highlights significant disparities in the screening and management of GDM in the south of Morocco, revealing persistent challenges and opportunities for improvement.

**FIGURE 2:**
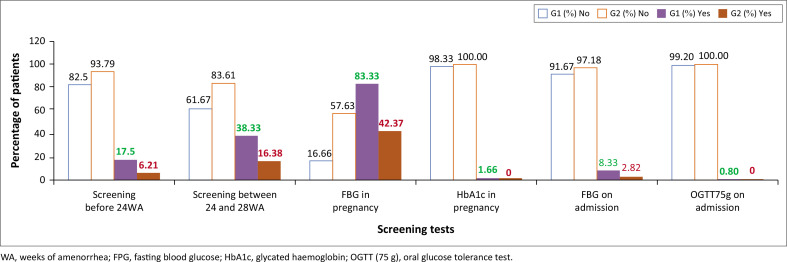
Rate of screening tests among both groups of pregnant women.

Early screening before 24 WA was performed in only 17.5% (95% CI: 11.5% – 25.6%) of patients (Figure 2), falling far below national recommendations.^17^

This study underscores the inadequacy of therapeutic management for pregnant women. In fact, screening before 24 weeks (WA) was conducted in only 17.50 of women diagnosed with GDM. This initial diagnostic delay contrasts with the 38.3% screening rate observed between 24 weeks and 28 weeks of WA (Figure 2), which significantly exceeds the 21% reported in an earlier Moroccan study.^5,18^ While this improvement suggests better adherence to protocols during routine second-trimester visits, critical gaps remain.

This cohort (*n* = 120) exhibited classic GDM risk factors, including older maternal age (32.6 ± 6.4 years) and obesity (65.3% with BMI ≥ 30) (Table 1). Despite their high-risk profile, these women did not receive sufficiently early or systematic screening. Therapeutically, 47.5% of patients achieved glycaemic control through diet and lifestyle modifications alone, while 17.5% required insulin therapy (Figure 2). These findings confirm the efficacy of nutritional interventions as first-line therapy.^19^ Most pregnant women are able to successfully normalise their blood sugar levels.

**TABLE 1:** Description of study population.

Variables	Gestational diabetes				Without gestational diabetes			
	Avg	s.d.	Freq	%	Avg	s.d.	Freq	%
Age of pregnancy (weeks)	38.50	2.05	-	-	38.50	2.39	-	-
Age of the woman (years)	32.63	6.36	-	-	28.63	6.00	-	-
**Marital status**								
Single	-	-	6	66.67	-	-	3	33.33
Married	-	-	114	39.58	-	-	174	60.42
**Provenance**								
Rural	-	-	73	41.71	-	-	102	58.29
Urban	-	-	47	38.52	-	-	75	61.48
**BMI (kg/m^2^)**								
18.5–24.9	-	-	7	11.29	-	-	55	88.71
25–29.9	-	-	49	35.77	-	-	88	64.23
≥ 30	-	-	64	65.31	-	-	34	34.69
**Parity**								
1	-	-	53	40.77	-	-	77	59.23
2	-	-	35	36.46	-	-	61	63.54
3	-	-	22	45.83	-	-	26	54.17
4	-	-	7	43.75	-	-	9	56.25
5	-	-	1	25.00	-	-	3	75.00
6	-	-	0	0.00	-	-	1	100.00
7	-	-	2	100.00	-	-	0	0.00
**Number of pregnancies**								
1	-	-	32	31.68	-	-	69	68.32
2	-	-	30	36.59	-	-	52	63.41
3	-	-	32	57.14	-	-	24	42.86
4	-	-	13	39.39	-	-	20	60.61
5	-	-	5	50.00	-	-	5	50.00
6	-	-	4	44.44	-	-	5	55.56
7	-	-	4	100.00	-	-	0	0.00
8	-	-	0	0.00	-	-	2	100.00
**Medical history**								
Yes	-	-	36	63.16	-	-	21	36.84
No	-	-	84	35.00	-	-	156	65.00
**Obstetrical history**								
Yes	-	-	66	58.93	-	-	46	41.07
No	-	-	54	29.19	-	-	131	70.81
**History of surgery**								
Yes	-	-	21	56.76	-	-	16	43.24
No	-	-	99	38.08	-	-	161	61.92
Length of hospital stay	3.23	-	1.05	2.45	-	-	0.75	-
**Pregnancy follow-up**								
Yes	-	-	80	40.82	-	-	116	59.18
No	-	-	40	39.60	-	-	61	60.40
Uterine height	32.56	-	3.38	31.74	-	-	2.46	-
**Termination of pregnancy**								
Yes	-	-	14	100.00	-	-	0	0.00
No	-	-	106	37.46	-	-	177	62.54
**Gestational hypertension**								
Yes	-	-	22	66.67	-	-	11	33.33
No	-	-	98	37.12	-	-	166	62.88
**TPD**								
Yes	-	-	7	43.75	-	-	9	56.25
No	-	-	113	40.21	-	-	168	59.79
**PROM**								
Yes	-	-	32	42.67	-	-	43	57.33
No	-	-	88	39.64	-	-	134	60.36
**Preeclampsia**								
Yes	-	-	13	81.25	-	-	3	18.75
No	-	-	107	38.08	-	-	174	61.92

s.d., standard deviation; Avg, average; Freq, frequency; BMI, body mass index; ATCD, antecedents; TPD, threatened preterm delivery; PROM, premature rupture of membranes.

However, in approximately 30% of patients inadequate response necessitates a step-up to second-line therapy, notably when glycaemic imbalances are identified.^11,20^ At hospital admission, only 8.3% of women with GDM received fasting blood glucose testing. This represents a critical care gap, as admission glucose monitoring serves as a key intervention for hyperglycaemia detection. Current evidence demonstrates that blood glucose levels > 120 mg/dL during labour warrant intrapartum insulin therapy.^21^ Furthermore, even mild untreated hyperglycaemia has been associated with a 1.8-fold increased risk of caesarean delivery^22^ underscoring the importance of systematic glucose surveillance in this population.

### During the postpartum period

**FIGURE 3:**
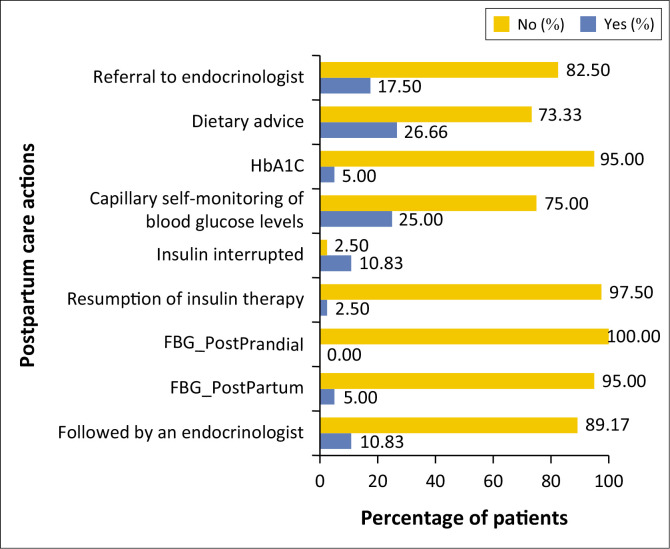
Postpartum care of women with gestational diabetes.

Our study found that only 10.83% of women who delivered with gestational diabetes were followed by an endocrinologist, and fasting blood glucose testing was requested for only 5% of these women (Figure 3). These findings align with various studies indicating that postpartum screening is notably low and infrequent.^23,24^ However, women with this pathology have a significantly increased risk (OR = 17.92) of developing type 2 diabetes.^11,25,26^

In addition, 10% of women who have had gestational diabetes develop diabetes immediately after delivery.^27^ Therefore, monitoring blood glucose levels in the immediate postpartum period is crucial for improving follow-up rates for women with GDM.^26^ Implementing a reminder system for check-ups and educational initiatives could also help mitigate future risks for these women.^24^

Regarding capillary self-monitoring of blood glucose levels, the study found that only 25% of women who delivered with gestational diabetes performed this monitoring (Figure 2). This lack of postpartum monitoring may stem from insufficient knowledge and awareness about the importance of monitoring during this period to detect hyperglycaemia associated with GDM. Similarly, a study by Balaji et al. emphasised that this deficiency is largely related to emotional stress and difficulties in adapting to motherhood among new mothers.^23^

Out of the 120 pregnant women with GDM in our sample, only 32 received dietary advice (Figure 2). Adhering to a healthy diet for all women with GDM can reduce the risk of developing type 2 diabetes and cardiovascular disease by 30%.^28,29^

In this regard, the Moroccan healthcare system has yet to establish strategies for regular postpartum follow-up, especially for women who have had gestational diabetes. To address this, developing a telephone support system and implementing telemedicine consultations, particularly for educational purposes,^28^ along with leveraging artificial intelligence, are key recommendations to ensure proper follow-up for women after delivery.

### Obstetrical management during labour and delivery

Various studies have indicated that the risk of caesarean delivery is elevated in women with GDM.^24,30^ Our study demonstrated a significant association between caesarean delivery and GDM, with aOR = 3.42 (95% CI: 2.07–5.66; *p* = 0.007) than the comparison group (Table 2). In addition, a logistic regression model was utilised to assess the relationship between GDM and maternal complications at delivery (Table 3). The findings revealed that the increased risk of caesarean delivery among women with GDM was 2.52 (95% CI: 1.29–4.92) (Table 3). The primary reason for caesarean sections was macrosomia. occurring in 76.32% of cases (aOR: 5.94; 95% CI: 2.70–13.11; *p* < 0.001) (Table 2). These results align with a case-control study by Luhete et al.^31^, which reported that caesarean delivery for macrosomia occurred in 15.6% of cases compared to 9.8% in controls, yielding a twofold increased risk for macrosomia (adjusted OR = 1.7; 95% CI: 1.0–2.8; *p* = 0.0419).^31^

**TABLE 2:** Obstetrical complications during labour and delivery in the two groups of pregnant women with and without gestational diabetes mellitus.

Obstetrical complications	DG = Yes (1st group)				DG = No (2nd group)				*p* (Chi^2^)	*p* (*F*)
	*n*	%	aOR	95% CI	*n*	%	aOR	95% CI		
**Complications during labour**										
Starting work	33	47.83	1.480	0.86–2.56	36	52.17	0.6731	0.39–1.16	0.1957	-
Premature rupture of membranes	32	42.67	1.130	0.67–1.93	43	57.33	0.8800	0.52–1.50	0.7446	-
Antepartum haemorrhage	2	40.00	0.980	0.16–5.97	3	60.00	1.0200	0.17–6.18	-	1.0000
**Labour complications**										
Pregnancy-induced hypertension	22	66.67	3.390	1.57–7.28	11	33.33	0.2900	0.14–0.63	0.0021	-
Suspicion of chorioamnionitis	1	16.67	0.290	0.03–2.51	5	83.33	3.4600	0.40–29.99	-	0.4069
Foetal heart rate anomaly	29	50.88	1.700	0.95–3.03	28	49.12	0.5900	0.33–1.05	0.1005	-
Tinted amniotic fluid	24	45.28	1.260	0.70–2.32	29	54.72	0.7800	0.43–1.43	0.5195	-
Foetal suffering	26	52.00	1.760	0.96–3.25	24	48.00	0.5700	0.31–1.04	0.0941	-
Threat of premature delivery	7	43.75	1.160	0.42–3.19	9	65.25	0.8600	0.31–2.39	0.9852	-
**Caesarean section reasons**										
Macrosomia	29	76.32	5.950	2.70–13.11	9	23.68	0.1700	0.08–0.37	< 0.0010	-
Preeclampsia	13	81.25	7.050	1.96–25.30	3	18.75	0.1400	0.04–0.51	0.0016	-
Hydramnios	5	100.00	-	0	0	-	-	-	-	-
Foetal heart rate abnormality	29	49.15	1.510	0.86–2.70	30	50.85	0.6600	0.37–1.17	0.1989	-
Gestational hypertension	7	77.78	5.300	1.08–25.96	2	22.22	0.1900	0.04–0.92	0.0527	-
**Delivery methods**										
Normal non-instrumental delivery (VB)	30	28.57	0.450	0.27–0.75	75	71.43	2.2100	1.32–3.67	0.0032	-
Caesarean section (upper approach)	60	60.00	3.420	2.07–5.66	40	40.00	0.2900	0.18–0.48	< 0.0010	-
Normal delivery by instrumental extraction	31	32.63	0.610	0.37–1.02	64	67.37	1.6200	0.98–2.71	0.0809	-
**Dystocia at the time of delivery**										
Tight circular umbilical cord	18	56.25	2.004	0.9549–4.21	14	43.75	0.5000	0.24–1.05	0.0942	-
Shoulder dystocia	15	88.24	12.21	2.74–54.48	2	11.76	0.0800	0.02–0.36	< 0.001	-
**Types of delivery**										
Spontaneous delivery (normal)	60	31.41	0.350	0.21–0.57	131	68.59	2.8500	1.74–4.65	< 0.0010	-
Premature delivery	11	37.93	0.870	0.39–1.91	18	62.07	1.1500	0.52–2.53	0.8807	-
Scheduled delivery	15	65.22	3.020	1.24–7.36	8	34.78	0.3300	0.14–0.81	0.0212	-
**Perineal trauma**										
Episiotomy	42	31.82	0.520	0.32–0.84	90	68.18	1.9200	1.19–3.10	0.0099	-
Perineal tear	15	62.50	2.600	1.10–6.16	9	37.50	0.3800	0.16–0.91	0.0430	-
First-degree tear	2	50.00	1.450	0.20–10.43	2	50.00	0.6900	0.10–4.97	-	1.0000
Uncomplicated complete perineal tear	0	0.00	0.000	1	100	-	-	-	-	1.0000
**Immediate postpartum (48 h)**										
Early breastfeeding	46	34.07	0.610	0.38–0.98	89	65.93	1.6300	1.01–2.61	0.0560	-
Immediate postpartum haemorrhage	13	59.09	2.270	0.94–5.49	9	40.91	0.4400	0.18–1.071	0.1030	-
Puerperal infection	16	50.00	1.550	0.74–3.23	16	50.00	0.6500	0.31–1.35	0.3269	-

GDM, gestational diabetes mellitus; aOR, adjusted odds ratio; CI, confidence interval; VB, vaginal birth.

**TABLE 3:** Logistic regression analysis of pregnancy outcomes associated with gestational diabetes mellitus.

Variables	OR	95% CI	*P*
Caesarean section	2.52	1.29–4.92	0.007
Complications during labour	1.38	0.702–2.693	0.352
Preeclampsia	5.95	1.21–29.21	0.028
Shoulder dystocia	16.30	3.32–79.95	0.001
Perineal tear	3.04	1.74–5.28	< 0.001
Immediate postpartum haemorrhage	1.82	0.66–5.01	0.246
Puerperal infection	1.59	0.68–3.73	0.284

OR, odds ratio; CI, confidence interval.

From another perspective, all study findings consistently indicate a strong association between GDM and preeclampsia.^14,32^

To enhance our statistical analysis, a logistic regression model was employed to examine the relationship between GDM and predictor characteristics related to pregnancy outcomes in our study population. The analysis confirmed that gestational diabetes was significantly associated with several other delivery outcomes: preeclampsia (OR = 5.95; 95% CI: 1.21–29.21), shoulder dystocia (OR = 16.30; 95% CI: 3.32–79.95) and perineal tear (OR = 3.04; 95% CI: 1.74–5.28). The magnitude of the associated risk is particularly striking for shoulder dystocia and preeclampsia. Conversely, while trends towards increased risk were noticed for general complications during labour, PPH and puerperal infection, these associations did not reach statistical significance in this study (Table 3).

According to several international studies, macrosomia is one of the primary factors linked to perineal tears during childbirth.^33^

Regarding immediate PPH, this study indicated a potentially elevated risk for women with GDM (OR = 1.82; 95% CI: 0.66–5.01); however, this association did not reach statistical significance (Table 3). This finding corroborates the results reported by Ye et al.,^34^ who similarly found no statistically significant association between gestational diabetes and the risk of immediate PPH.

### Gestational diabetes mellitus risk factors

In another aspect, this study examined factors that may be associated with GDM among women from southern Morocco. The analysis confirmed maternal age as a significant risk factor for GDM (β = 0.076; OR = 1.08; 95% CI: 1.03–1.13; *p* < 0.001), indicating that each additional year increases the odds by about 7.9%, consistent with age-related declines in insulin sensitivity (Table 5). This aligns with other studies that recognise advanced maternal age (typically > 35 years) as a key risk factor for developing GDM.^12,35^

Obesity (BMI ≥ 30 kg/m^2^) was identified as a strong risk factor for GDM (*p* < 0.001), affecting 65.3% of women in this category. Compared to normal weight individuals, obese women exhibited significantly higher odds – nearly five times greater – of developing GDM (OR = 4.81; 95% CI: 2.86–8.07) (Table 4). These findings align with a study conducted in Egypt, where 50% of women with GDM had a BMI greater than 30 (*p* < 0.001), compared to 21.2% of women without GDM. Thus, obesity is recognised as a major risk factor for the development of GDM.^12,27^ These results underscore the importance of weight management in pregnant women to help prevent both maternal and foetal complications associated with GDM.

**TABLE 4:** Risk factors for gestational diabetes mellitus in the women studied.

Clinical and sociodemographic Characteristics	GDM = Yes (1st group)				GDM = No (2nd groups)				*p* (Chi^2^)
	*n*	%	aOR	95% CI	*n*	%	aOR	95% CI	
**Provenance**									
Agadir	34	36.56	0.79	0.47–1.31	59	63.44	1.26	0.76–2.10	0.4329
Biougra	31	49.21	1.58	0.90–2.76	32	50.79	0.63	0.36–1.11	0.1445
Inezgane	22	34.92	0.74	0.42–1.33	41	65.08	1.34	0.75–2.40	0.3928
Taroudannt	33	42.31	1.11	0.66–1.88	45	57.69	0.90	0.53–1.52	0.7913
**Environment**									
Urban	47	38.52	0.88	0.54–1.40	75	61.48	1.14	0.71–1.83	0.6666
Rural	73	41.71	1.14	0.71–1.83	102	58.29	0.87	0.55–1.40	0.6666
**Medical history**									
Gestational hypertension	15	83.33	8.29	2.34–29.30	3	16.67	0.12	0.03–0.43	0.0003
Family history (type 2 diabetes)	13	92.86	21.38	2.76–165.79	1	7.14	0.05	0.006–0.36	0.0001
**History of surgery**									
Appendectomy	7	70.00	3.52	0.91–14.18	3	30.00	0.28	0.07–1.10	0.1069
Gall bladder	14	60.87	2.46	1.03–5.90	9	39.13	0.40	0.17–0.97	0.0627
**Obstetrical history**									
History of abortion	35	55.56	2.19	1.25–3.85	28	44.44	0.46	0.26–0.80	0.0089
History of foetal death in utero	9	60.00	2.31	0.80–6.67	6	40.00	0.43	0.15–1.25	0.1878
History of prematurity	15	68.18	3.47	1.36–8.79	7	31.82	0.28	0.11–0.73	0.0113
History of stillbirths	6	37.50	0.88	0.31–2.49	10	100.00	1.14	0.40–3.22	1.0000
History of macrosomia	16	84.21	8.92	2.54–31.36	3	15.79	0.11	0.03–0.39	0.0002
**BMI (kg/m^2^)**									
18.5 kg/m^2^ – 24.9 kg/m^2^: normal weight	7	11.29	0.14	0.06–0.31	55	88.71	7.28	3.18–16.64	< 0.001
25 kg/m^2^ – 29.9 kg/m^2^: overweight	49	35.77	0.70	0.44–1.11	88	64.23	1.43	0.90–2.29	0.1650
≥ 30 kg/m^2^: obesity	64	65.31	4.81	2.86–8.07	34	34.69	0.21	0.12–0.34	< 0.001

GDM, gestational diabetes mellitus; aOR, adjusted odds ratio; CI, confidence interval; BMI, body mass index.

However, the logistic regression model (Table 5) reports, for overweight women, β = –2.530 (OR = 0.08; *p* < 0.001) translates to a 92% risk reduction. For obesity, β = –1.075 (OR = 0.34; *p* < 0.001) corresponds to a 66% risk reduction. These negative β values, while counterintuitive, show statistical robustness and may suggest the presence of specific protective factors in this population or differences in medical management.

**TABLE 5:** Logistic regression analysis of associated factors with gestational diabetes.

Risk factors	OR	95% CI	*P*
Age of the women (years)	1.08	1.03–1.13	< 0.001
BMI (Ref. = normal weight)	-	-	< 0.001
BMI (Ref. = overweight)	0.08	0.03–0.21	< 0.001
BMI (Ref. = obesity)	0.34	0.19–0.62	< 0.001
Family History (Ref. = No)	0.05	0.01–0.53	0.013
History of abortion (Ref. = No)	0.90	0.45–1.79	0.765
History of macrosomia (Ref. = No)	0.24	0.06–1.01	0.052
History of prematurity (Ref. = No)	0.34	0.12–0.97	0.044

BMI, body mass index; Ref., reference; No, number; OR, odds ratio; CI, confidence interval.

Another factor examined was the family history of type 2 diabetes. The study found an aOR = 21.38 (95% CI: 2.76–165.79), indicating a significant association between this factor and GDM (*p* = 0.0001) (Table 4). This finding aligns with previous research that highlights family history of type 2 diabetes as one of the most common risk factors for GDM.^36^

Furthermore, this study found a significant association between a history of abortion and GDM, with aOR = 2.19 (95% CI: 1.25–3.85; *p* < 0.0089). Similarly, there was a significant association between a history of prematurity and GDM, with an aOR = 3.47 (95% CI: 1.37–8.79; *p* < 0.0113) (Table 4). These findings align with a study conducted in Jordan, which also reported significant associations between both a history of abortion and prematurity with GDM.^35^ This study showed an association between a history of macrosomia and GDM compared to pregnant women without GDM, with aOR= 8.92 (95% CI: 2.54–31.36 vs. aOR = 0.11 [95% CI: 0.03–0.39; *p* = 0.0002]).

According to the regression model, obstetric history reveals notable variations. Preterm birth shows β = –1.080 (OR = 0.34; *p* = 0.044), corresponding to a 66% risk reduction. Macrosomia demonstrates β = –1.433 (OR = 0.24; *p* = 0.052), equivalent to a 76% reduction, although this association approaches but does not reach conventional statistical significance. History of abortion shows a non-significant β (–0.105; *p* = 0.765), indicating no clear association with gestational diabetes in this study.

## Conclusion

This study, conducted in the Souss-Massa region, underscores the critical need for localised strategies to address GDM in southern Morocco. The findings highlight significant deficiencies in current GDM management practices, particularly concerning suboptimal antenatal screening and the near absence of structured postpartum follow-up. This leaves women vulnerable to adverse delivery outcomes and increased risks for future type 2 diabetes, thereby emphasising the substantial clinical impact of unaddressed GDM within this population.

Consequently, there is an urgent imperative to improve GDM screening and follow-up care in the region. Recommendations include strengthening antenatal screening protocols, incorporating standardised diagnostic tests, and establishing dedicated, multidisciplinary postpartum care pathways that include robust patient education. Future research should focus on identifying local barriers (cultural, logistical and informational) to accessing recommended care, and exploring targeted interventions, such as telehealth, to improve adherence. Furthermore, longitudinal studies evaluating the long-term consequences of GDM on the health of mothers and children from these pregnancies in the specific context of Souss-Massa are needed, as are health economic analyses to guide resource allocation towards the most efficient management strategies.
